# Performance of High-Resolution Melting Analysis for Molecular Detection and Species Differentiation of Malaria: A Scoping Review

**DOI:** 10.3390/pathogens15080815

**Published:** 2026-08-01

**Authors:** Afiat Berbudi, Shafia Khairani, Andromeda Andromeda, Alexander Kwarteng

**Affiliations:** 1 Department of Biomedical Sciences, Parasitology Division, Faculty of Medicine, Universitas Padjadjaran, Bandung 40161, Indonesia; 2 Research Center for Care and Control of Infectious Diseases (RC3ID), Universitas Padjadjaran, Bandung 40161, Indonesia; 3 Department of Biomedical Sciences, Cell Biology Division, Faculty of Medicine, Universitas Padjadjaran, Bandung 40161, Indonesia; shafia@unpad.ac.id; 4 Veterinary Medicine Program, Faculty of Medicine, Universitas Padjadjaran, Sumedang 45363, Indonesia; 5 Departement of Biomedical Sciences, Parasitology Division, Faculty of Medicine, Pasundan University, Bandung 40117, Indonesia; andromeda@unpas.ac.id; 6 Department of Biochemistry and Biotechnology, Kwame Nkrumah University of Science and Technology, PMB University Post Office, Kumasi, Ghana; akwarteng@knust.edu.gh

**Keywords:** malaria, *Plasmodium*, high-resolution melting, HRM, molecular diagnosis

## Abstract

Malaria diagnosis remains challenging in low-density, asymptomatic, mixed-species, and non-falciparum infections. This scoping review mapped the diagnostic and operational evidence for high-resolution melting (HRM) analysis in malaria. Following PRISMA-ScR guidance, PubMed and Scopus were searched for English-language studies published between 2016 and 2026. Two reviewers independently screened records in Rayyan. Of 162 records identified, 15 studies met the eligibility criteria. Most assays targeted 18S rRNA, although mitochondrial, apicoplast, species-specific, and resistance-associated markers were also evaluated. Reported sensitivity ranged from 91.76% to 100%, and specificity generally exceeded 92%. HRM enabled differentiation of major human-infecting *Plasmodium* species and detected mixed, low-parasitemia, and submicroscopic infections that were missed by microscopy or rapid diagnostic tests. However, substantial heterogeneity in assay targets, platforms, comparators, and reporting limited direct comparison. Current applications remain laboratory-developed and require HRM-capable real-time PCR instruments, dedicated analytical software, and trained personnel. HRM is therefore a promising complementary method for reference laboratories and malaria surveillance, but standardized assays, multicentre validation, and implementation studies are required before routine or decentralized use.

## 1. Introduction

Malaria remains one of the most important parasitic diseases worldwide and continues to impose a substantial public health burden in endemic regions. According to the World Malaria Report 2025, there were an estimated 282 million malaria cases and 610,000 malaria deaths globally in 2024, indicating a continued increase in the global malaria burden compared with the previous year. Despite substantial progress since 2000, including an estimated 2.3 billion cases and 14 million deaths averted, malaria remains a persistent global health challenge, particularly in the WHO African Region, where the greatest share of cases and deaths continues to occur [1]. Accurate and timely diagnosis is central to effective case management, treatment allocation, and surveillance, particularly in areas moving toward elimination where low-density and asymptomatic infections may sustain residual transmission. WHO recommends prompt parasitological confirmation by microscopy or rapid diagnostic tests (RDTs) for all patients with suspected malaria before treatment initiation, emphasizing the importance of reliable diagnostic tools for both clinical care and surveillance systems [1].

Conventional malaria diagnosis relies mainly on light microscopy and RDTs. Microscopy remains useful because it can identify parasites, estimate parasite density, and differentiate species when performed by skilled personnel. However, its performance is highly operator-dependent and may be limited in low-parasitemia infections, mixed-species infections, and settings with limited laboratory quality assurance [2]. RDTs are operationally simpler and widely used in endemic areas, but they provide limited species resolution and may miss infections with low antigen levels, non-falciparum species, or parasite variants that affect target antigen expression [3]. These limitations are particularly important in elimination settings, where asymptomatic and submicroscopic infections may remain undetected by conventional diagnostic approaches. WHO notes that early and accurate diagnosis is essential not only for patient management but also for strong malaria surveillance.

Molecular diagnostic methods offer greater analytical sensitivity than microscopy and RDTs and are increasingly used for species confirmation, detection of submicroscopic infections, and epidemiological surveillance [4]. Among these methods, high-resolution melting (HRM) analysis has emerged as a promising post-amplification technique that combines real-time PCR amplification with melting curve analysis in a closed-tube format [5,6]. HRM detects sequence variation based on differences in melting temperature and curve profiles, enabling *Plasmodium* detection, species differentiation, and, in some assays, the identification of mixed infections or genetic variants [6]. Compared with conventional nested PCR, HRM may reduce contamination risk [7,8], eliminate gel electrophoresis [9,10], shorten turnaround time [11,12,13], and lower costs when intercalating dyes are used instead of species-specific probes or sequencing-based confirmation [10,12,13].

Several studies have evaluated HRM-based assays for malaria diagnosis using different molecular targets and platforms. The 18S small subunit ribosomal RNA gene has been frequently used because it allows pan-*Plasmodium* detection and species discrimination through conserved and variable regions [8,10,11,14]. Other assays have targeted mitochondrial genes, such as cytochrome b and cox1, or organellar targets such as clpC to improve sensitivity or resolve closely related species and subspecies [13,15,16]. HRM has also been applied to differentiate major human-infecting *Plasmodium* species, including *P. falciparum*, *P. vivax*, *P. malariae*, *P. ovale*, and *P. knowlesi*, and to distinguish *P. ovale curtisi* from *P. ovale wallikeri* [9,13,16].

Despite these promising applications, the evidence on HRM for malaria diagnosis remains fragmented. Existing studies vary substantially in geographical setting, sample type, target gene, HRM platform, comparator method, and reported diagnostic outcomes [5,6]. Some studies focus on assay development and analytical validation, whereas others evaluate HRM in clinical, retrospective, or field-based surveillance contexts. Differences in reference standards, such as microscopy, RDTs, nested PCR, LAMP, TaqMan qPCR, or sequencing, further complicate direct comparison of diagnostic performance across studies [4,17,18]. Moreover, the role of HRM in detecting low-parasitemia infections, resolving natural mixed infections, identifying non-falciparum species, and supporting routine or field-based surveillance has not been comprehensively mapped.

Given the limited number of primary studies and the methodological heterogeneity across HRM applications, including differences in molecular targets, assay designs, reference methods, and study populations, a scoping review provides an appropriate framework to systematically map the available evidence and identify current knowledge gaps [19,20]. Rather than estimating pooled diagnostic accuracy, this review aims to synthesize the breadth and characteristics of the existing evidence by examining how HRM has been applied for malaria diagnosis, species differentiation, mixed-infection detection, and the identification of low-parasitemia infections. Specifically, this scoping review evaluates the reported diagnostic and analytical performance of HRM in human clinical samples while identifying methodological variations, operational considerations, and evidence gaps that may inform future research and implementation.

## 2. Methods

### 2.1. Study Design and Reporting Framework

This study was conducted as a scoping review to map the available evidence on the application of high-resolution melting (HRM) analysis for the molecular diagnosis of malaria. A scoping review was considered appropriate because the objective was to characterize the existing evidence, summarize the reported applications and diagnostic performance of HRM, and identify knowledge gaps across heterogeneous study designs, molecular targets, and laboratory protocols rather than to estimate pooled diagnostic accuracy.

The review was conducted and reported in accordance with the Preferred Reporting Items for Systematic Reviews and Meta-Analyses Extension for Scoping Reviews (PRISMA-ScR) and the methodological guidance of the Joanna Briggs Institute (JBI) [19,20]. The study protocol was prospectively registered in the Open Science Framework (OSF; DOI: 10.17605/OSF.IO/47AKE).

**Figure 1:**
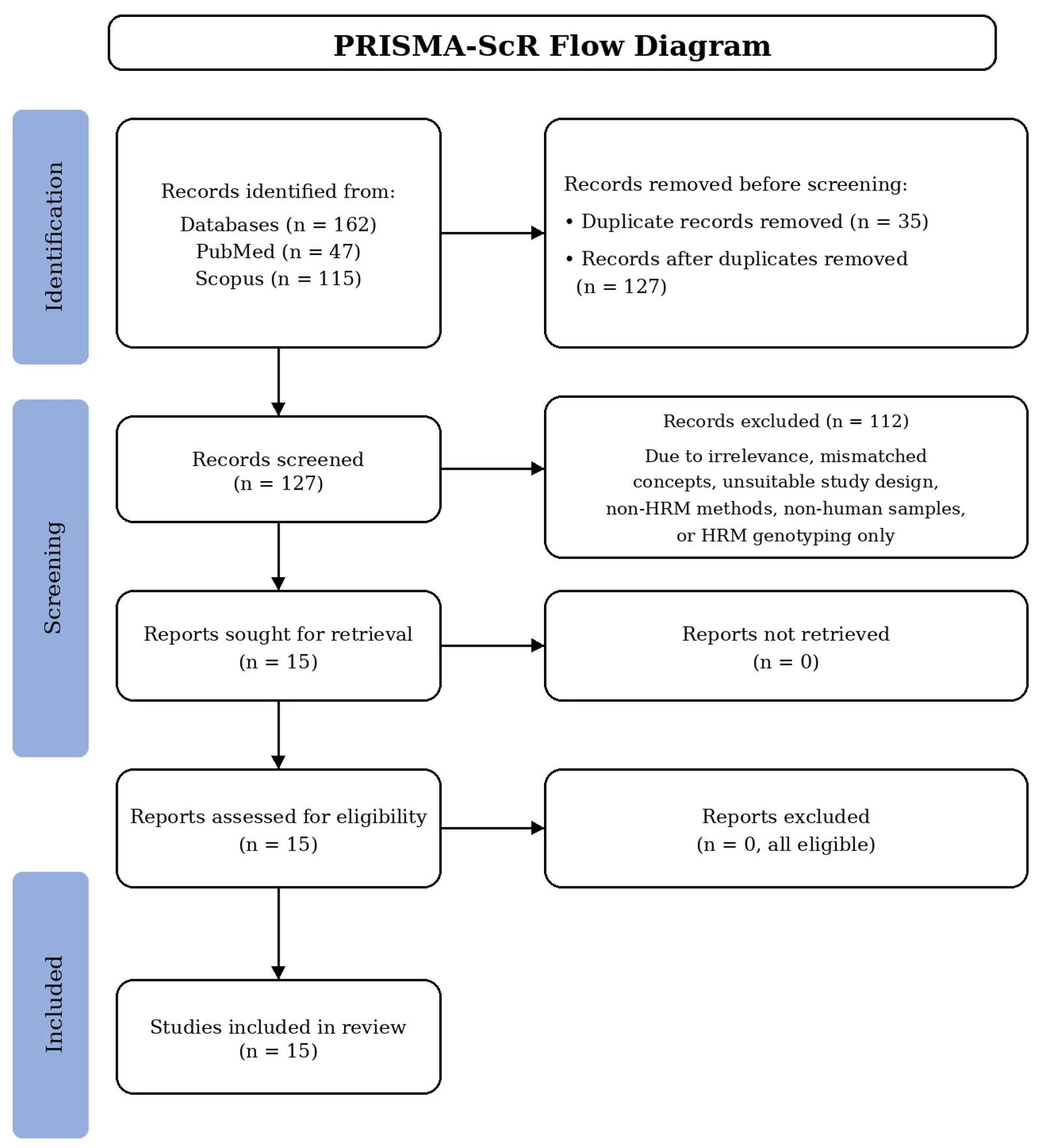
PRISMA flow diagram of the study selection process.

Study screening was performed independently by two reviewers using the Rayyan web platform (Rayyan Systems Inc., Cambridge, MA, USA) with blinded decision-making. Disagreements were resolved through discussion until consensus was reached. The study selection process is presented in the PRISMA flow diagram (Figure 1).

### 2.2. Review Question and PEO Framework

The review was guided by the following research question:

“What is the reported diagnostic and analytical performance of high-resolution melting (HRM) analysis for the molecular detection and species differentiation of malaria in human clinical samples?”

The review scope was defined using the Population–Exposure–Outcome (PEO) framework. The population comprised human clinical blood samples from individuals with suspected or confirmed malaria. The exposure was HRM-based molecular diagnostic assays, and the outcomes included reported diagnostic performance, *Plasmodium* species differentiation, detection of mixed or low-parasitemia infections, and relevant analytical and operational characteristics.

### 2.3. Eligibility Criteria

Studies were considered eligible if they evaluated HRM-based molecular assays for malaria diagnosis using human blood-derived clinical samples. Eligible outcomes included at least one of the following: sensitivity, specificity, accuracy, limit of detection, agreement with comparator methods, species differentiation, detection of mixed infections, detection of low-parasitemia or submicroscopic infection, or analytical and operational characteristics.

Studies were included if they were original diagnostic or analytical studies published in English between 2016 and 2026. Studies were excluded if they did not use HRM-based assays, did not involve human blood samples, did not evaluate malaria or *Plasmodium* infection, or reported HRM only for genotyping purposes without diagnostic relevance. Reviews, editorials, commentaries, conference abstracts without sufficient original data, and non-English studies were also excluded.

### 2.4. Information Sources and Search Strategy

A systematic literature search was conducted in PubMed/MEDLINE (U.S. National Library of Medicine, Bethesda, MD, USA) and Scopus (Elsevier B.V., Amsterdam, the Netherlands). The search strategy combined terms related to malaria, *Plasmodium*, HRM analysis, diagnosis, detection, identification, and differentiation. Searches were limited to publications from 2016 to 2026 and to English-language articles. The complete database-specific search strings, applied limits, and numbers of records retrieved are provided in Supplementary Table S1.

Search results from both databases were exported and screened. Rayyan web application was used to support the screening, deduplication, and reviewer decision process [21].

### 2.5. Study Selection Process

All records retrieved from PubMed and Scopus were imported into Rayyan. Duplicate records were identified and removed before screening. The title and abstract screening was conducted independently by two reviewers according to the predefined eligibility criteria. Studies considered irrelevant by both reviewers were excluded. Any disagreement between reviewers was resolved through discussion until consensus was reached.

### 2.6. Data Charting and Extraction

Data were charted from the included studies using a standardized extraction framework aligned with the PEO structure and review objectives. Extracted information included author, year of publication, country or study setting, study design or aim, sample type, sample size, HRM target gene or marker, HRM platform, comparator method, *Plasmodium* species evaluated, diagnostic performance outcomes, mixed infection detection, low-parasitemia or submicroscopic detection, and analytical or operational characteristics.

Data charting focused on mapping the range of evidence rather than conducting a formal risk-of-bias assessment. This approach is consistent with scoping review methodology, which emphasizes describing the extent, distribution, and nature of available evidence.

### 2.7. Data Synthesis

Data synthesis was performed using a narrative thematic approach, consistent with the objectives of a scoping review. Following data charting, the included studies were grouped according to their primary diagnostic objectives and reported outcomes. Thematic domains were developed iteratively to organize evidence on diagnostic performance, species differentiation, mixed-infection detection, low-parasitemia detection, analytical and operational characteristics, and evidence gaps. The synthesis focused on mapping the range and characteristics of the available evidence rather than quantitatively comparing diagnostic accuracy across studies.

## 3. Results

### 3.1. PRISMA Flow Summary

The database search identified 162 records, comprising 47 from PubMed and 115 from Scopus. After removal of 35 duplicates, 127 unique records underwent title and abstract screening. Of these, 112 records were excluded because they did not meet the predefined eligibility criteria. The remaining 15 studies fulfilled the eligibility criteria and were included in the final synthesis and Table 1. Screening was conducted independently by two reviewers using Rayyan, and disagreements were resolved through discussion. The selection process is summarized in Figure 1.

### 3.2. Characteristics of Included Studies

Fifteen studies were included in this scoping review and synthesized narratively. The studies evaluated HRM analysis for malaria detection, *Plasmodium* species differentiation, mixed-infection detection, low-parasitemia identification, treatment monitoring, and assay optimization. The included studies were conducted across both malaria-endemic and non-endemic settings, including Uganda [10,22], South Africa [23], Afghanistan [24,25], Iran [7,11,26], Colombia [8], Thailand [13], France [9,27,28], and Austria [12,16]. This geographical diversity allowed assessment of HRM across different epidemiological contexts, including asymptomatic community-based infections, imported malaria cases, and clinically suspected malaria.

**Table 1:** Summary Characteristics of Included Studies.

Author (Year)	Country/Setting	Sample Type	Sample Size	Comparator Method	Main Diagnostic Focus
[16]	Austria; samples from Bangladesh and Ethiopia	Filter-paper blood spots	33	Microscopy, nested PCR	*P. ovale curtisi* and *P. ovale wallikeri* discrimination
[7]	Iran	Blood samples	81	Microscopy	Diagnostic validation
[27]	France	EDTA whole blood	133	Microscopy, nested PCR, RDT	Treatment efficacy monitoring
[23]	South Africa	Finger-prick DBS	985	RDT, sequencing	Asymptomatic malaria surveillance
[24]	Afghanistan	DNA banking cards/slides	296	Microscopy, semi-nested multiplex PCR	Asymptomatic malaria detection
[25]	Afghanistan	Thick and thin blood films	347	Microscopy	Mixed infection detection
[28]	France	EDTA whole blood	183	LAMP, microscopy, TaqMan qPCR	Molecular diagnostic validation
[8]	Colombia	FTA card blood samples	41	Microscopy, nested PCR	Assay development and species identification
[12]	Austria; imported samples	Archived filter papers	113	Microscopy, nested PCR	Species differentiation
[22]	Uganda	Venous blood/DBS	152	Microscopy, genomic controls	Species identification and resistance profiling
[26]	Iran	Peripheral blood/DNA cards/RDTs/slides	271	Microscopy, RDT, sequencing	Asymptomatic infection detection
[13]	Thailand	EDTA blood	120	Microscopy, qPCR, sequencing	Hexaplex assay validation
[9]	France	Fresh EDTA blood	356	Microscopy, nested PCR, TaqMan qPCR	*P. ovale* subspecies differentiation
[11]	Iran	Peripheral blood/slides	300	Microscopy, nested PCR, sequencing	Assay optimization and validation
[10]	Uganda	Giemsa-stained smears and red cell pellets	367	Microscopy	Sample-type analytical evaluation

The study designs included assay development and analytical validation [8,9,11,12,13,16], retrospective diagnostic comparisons [10,27,28], and cross-sectional field evaluations [7,22,23,24,25,26]. Clinical sample types varied substantially and included EDTA whole blood, peripheral blood, dried blood spots or filter-paper blood samples, blood stored on FTA or DNA banking cards, and archived Giemsa-stained thick blood smears or red blood cell pellets. Sample sizes ranged from 33 to 985 clinical samples. Comparator methods included microscopy, rapid diagnostic tests (RDTs), nested PCR, semi-nested multiplex PCR, LAMP, TaqMan qPCR, sequencing, and genomic controls. The main characteristics of the included studies are summarized in Table 1.

### 3.3. Molecular Targets and HRM Assay Designs

The included studies used diverse real-time PCR-HRM platforms and molecular targets. The most frequently targeted region was the 18S small subunit ribosomal RNA gene, which was used for pan-*Plasmodium* detection and species differentiation in several studies [7,8,9,10,11,24,26,27,28]. The diagnostic utility of 18S rRNA was attributed to its multi-copy nature and the presence of conserved regions flanking species-specific sequences, enabling amplification across *Plasmodium* species while generating distinguishable melting temperature profiles.

Other studies targeted mitochondrial and organellar markers, including cytochrome b, cox1, and the apicoplast clpC gene [12,13,16]. Mitochondrial targets were used to enhance sensitivity and support differentiation of human-infecting *Plasmodium* species, including *P. falciparum*, *P. vivax*, *P. malariae*, *P. ovale*, and *P. knowlesi*. Assay configurations varied from singleplex HRM assays to more complex multiplex approaches. One study developed a hexaplex HRM assay incorporating targets for five human *Plasmodium* species and an internal control [13], while another combined HRM with a TaqMan probe to improve confirmation of *P. falciparum* [12]. Some studies also used HRM for resistance-associated markers, including pfcrt and Pfk13/kelch13, although these were more relevant to molecular surveillance than primary diagnostic performance [22,23]. A study-level summary of the molecular markers, their intended applications, and principal analytical contributions is provided in Supplementary Table S2.

### 3.4. Diagnostic Performance of HRM

Overall, across the included studies, HRM was reported to achieve sensitivities ranging from 91.76% to 100%, while specificity generally exceeded 92% and reached 100% in several studies [9,11,13,24,28]. One cross-sectional study reported 100% sensitivity, 92.7% specificity, 100% positive predictive value, 92.7% negative predictive value, and 92.9% overall accuracy for HRM in detecting asymptomatic malaria [24]. Another study found that diagnostic performance depended on DNA source, with HRM using red blood cell pellets achieving 100% sensitivity and 94.0% specificity, whereas archived Giemsa-stained blood smears achieved 93.0% sensitivity and 96.7% specificity [10].

Agreement between HRM and molecular comparators was generally high. Kappa agreement ranged from moderate to near-perfect across studies, including 0.739 against nested PCR [8], 0.97 against LAMP [28], 1.00 in one hexaplex assay evaluation [13], and 0.999 against sequencing [11]. However, occasional false negatives and species-level misclassifications were reported. One study documented false negatives likely related to DNA loss or degradation during sample storage [22], while another reported species-level crossing reactions involving misidentification of *P. ovale* as *P. malariae* [9]. These findings indicate that HRM has strong diagnostic potential, but performance remains influenced by sample type, DNA quality, molecular target, reference standard, and species composition. A summary of the reported diagnostic performance metrics is presented in Table 2.

### 3.5. Species Differentiation and Mixed Infection Detection

HRM was consistently evaluated not only as a malaria detection tool but also as a method for differentiating *Plasmodium* species. Several assays successfully distinguished major human-infecting species, including *P. falciparum*, *P. vivax*, *P. malariae*, *P. ovale*, and *P. knowlesi*, based on distinct melting temperatures and curve profiles [8,12,13,28]. The hexaplex HRM assay generated multiple species-specific melting peaks and enabled simultaneous detection of five human *Plasmodium* species with an internal control [13]. Other assays focused on resolving closely related species or subspecies, particularly *P. ovale curtisi* and *P. ovale wallikeri* [9,12,16].

Several studies showed that HRM could detect mixed infections that were missed or underestimated by microscopy [7,8,12,23,24,25,28]. Reported mixed infections included *P. falciparum/P. vivax*, *P. falciparum/P. malariae*, and *P. falciparum/P. ovale*. In artificial mixing experiments, HRM detected minority species at ratios as low as 1:1000 [13]. Nevertheless, limitations were reported. Narrow melting-temperature differences, such as approximately 0.2 °C between some species or subspecies, could complicate interpretation [11,16]. In addition, multi-copy 18S rRNA variants within *P. falciparum* generated double-peak profiles that could mimic mixed infections [9,10]. Some assays also failed to detect minor species in natural clinical mixed infections or were designed only to distinguish *P. falciparum* from non-*falciparum* infections rather than identify all species present [9,28].

### 3.6. Low-Parasitemia and Submicroscopic Infection Detection

HRM showed strong potential for detecting low-parasitemia and submicroscopic malaria, particularly in asymptomatic populations and elimination settings. Reported limits of detection varied by assay target and platform. Assays targeting 18S rRNA reported limits of detection of approximately 2.35–3.31 copies/µL or around 6 copies/µL, corresponding to approximately 1 parasite/µL in some studies [8,13]. Other assays targeting mitochondrial or apicoplast markers reported limits of detection ranging from 8.90 to 46.43 copies/µL [12,16], while one assay differentiating *P. ovale curtisi* and *P. ovale wallikeri* reported detection down to 1 parasite/µL [9].

Several field studies demonstrated that HRM detected infections missed by microscopy or RDTs. In Afghanistan, HRM detected asymptomatic malaria prevalence of 8.78%, compared with 1.7% by microscopy [24]. In Iran, HRM identified asymptomatic infections in a cohort where both microscopy and RDTs detected no cases [26]. In Uganda, PCR-HRM detected *Plasmodium* DNA in microscopy-negative blood smears and red blood cell pellets, supporting its utility for identifying submicroscopic carriage [10]. HRM also detected persistent parasite DNA after treatment and identified low-parasitemia *P. falciparum* cases missed by nested PCR or commercial qPCR assays [11,27,28]. Collectively, these findings suggest that HRM may be valuable for surveillance and active case detection in low-transmission or pre-elimination settings, although evidence remains heterogeneous.

### 3.7. Analytical and Operational Considerations

The included studies consistently identified HRM as a rapid, closed-tube, post-PCR-free molecular method with operational advantages over conventional nested PCR and sequencing-based approaches. The closed-tube format reduces the risk of amplicon contamination and eliminates the need for gel electrophoresis or other post-amplification processing steps [7,8,9,11,13,28]. Where reported, assay turnaround time ranged from approximately 1 h and 15 min to 2 h [12,13]. HRM was also described as relatively cost-efficient compared with probe-based qPCR and sequencing, particularly when using intercalating dyes such as SYBR Green or EvaGreen [9,11,12,28]. One hexaplex HRM assay estimated the cost at approximately USD 4 per reaction [13].

Despite these advantages, HRM remains dependent on specialized real-time PCR instruments capable of precise thermal control and high-resolution fluorescence acquisition [11,13,16]. Accurate interpretation of melting curves also requires technical expertise, particularly when melting temperature differences are small or when complex multi-peak profiles occur [11]. The included studies therefore support HRM as a potentially useful method for reference laboratories, surveillance programs, and research settings, but not yet as a point-of-care diagnostic tool. None of the included studies explicitly evaluated HRM in a true point-of-care setting. The thematic distribution of evidence across the included studies is summarized in Table 3.

### 3.8. Evidence Gaps

Several evidence gaps were identified across the included studies. First, clinical validation remains limited by small sample sizes in some assay development studies, including cohorts of 33 and 41 clinical samples [8,16]. Although some studies were conducted in malaria-endemic settings, others relied on retrospective or imported malaria samples from non-endemic European reference laboratories [9,27,28]. This limits generalizability to routine diagnostic settings in endemic LMIC contexts.

**Figure 2:**
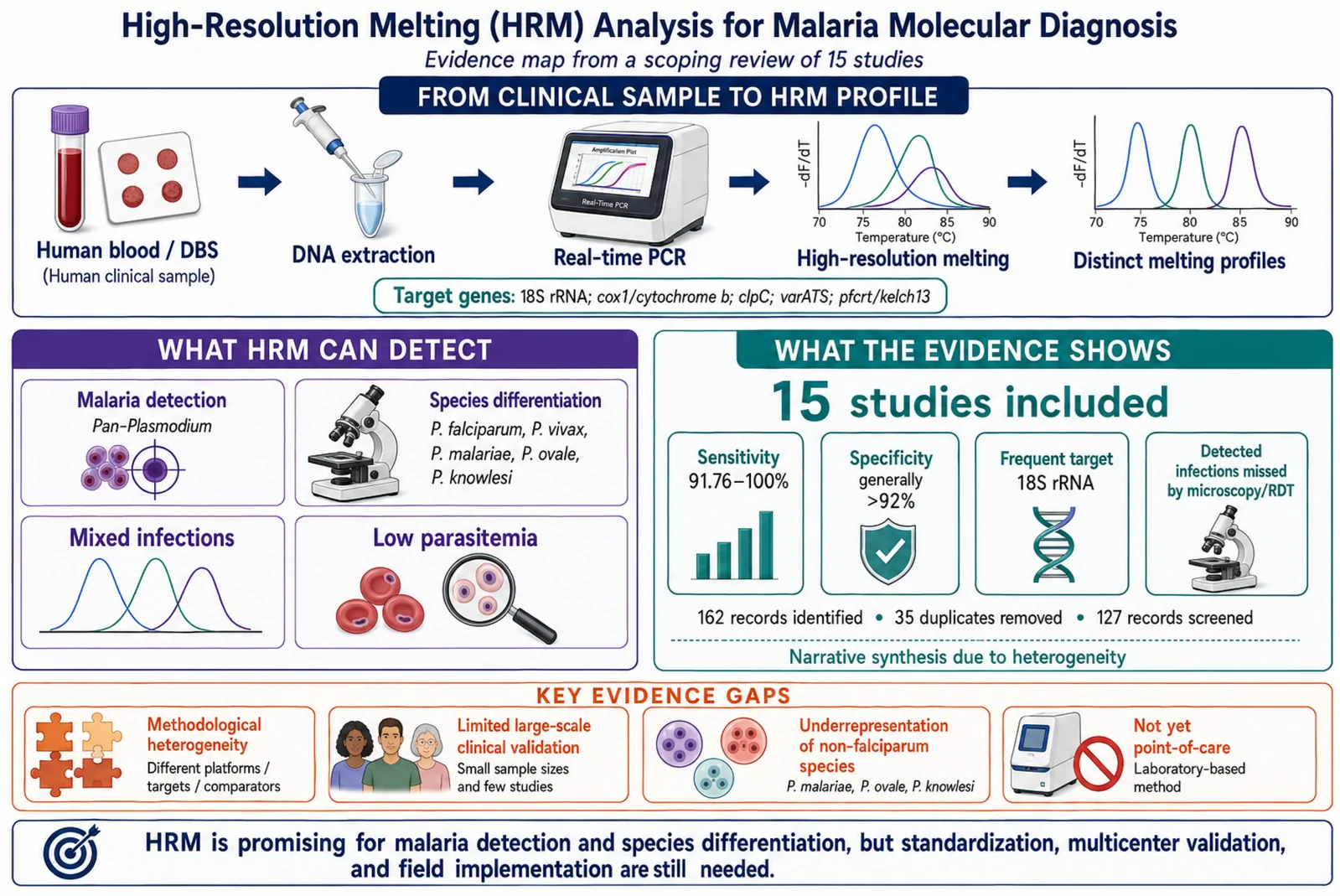
Evidence map of high-resolution melting (HRM) analysis for malaria molecular diagnosis. The figure summarizes the HRM workflow, from human blood or dried blood spot sampling through DNA extraction, real-time PCR amplification, and melting-profile analysis. Across the 15 included studies, HRM demonstrated potential for malaria detection, species differentiation, mixed-infection identification, and low-parasitemia detection, with reported sensitivity of 91.76–100% and specificity generally exceeding 92%. Key evidence gaps include methodological heterogeneity, limited large-scale clinical validation, underrepresentation of non-*falciparum* species, and the lack of point-of-care implementation. DBS, dried blood spot; PCR, polymerase chain reaction; RDT, rapid diagnostic test.

Second, non-*falciparum* species, mixed infections, and low-density infections remain underrepresented in clinical validation datasets. Species such as *P. malariae*, *P. ovale*, and *P. knowlesi* were often included in small numbers or assessed mainly through artificial mixtures or reference controls [9,11,13]. Third, there is no standardized HRM target, assay format, or melting temperature interpretation framework. Although 18S rRNA improves sensitivity, intra-genomic sequence variation can produce double-peak profiles and complicate species interpretation [9,10]. Finally, operational evidence remains limited. Direct field-based comparisons of HRM against RDTs, microscopy, commercial qPCR, and LAMP are still insufficient, and the feasibility of HRM for decentralized or point-of-care implementation remains unclear [11,13]. The key evidence gaps and their implications for future research are summarized in Table 4. The overall evidence map of HRM-based malaria diagnosis, including sample inputs, assay workflow, diagnostic outputs, key findings, and evidence gaps, is presented in Figure 2.

## 4. Discussion

### 4.1. Principal Findings

This scoping review mapped the available evidence on the use of high-resolution melting (HRM) analysis for the molecular diagnosis of malaria, with particular attention to diagnostic performance, *Plasmodium* species differentiation, detection of mixed infections, detection of low-parasitemia, and operational considerations. Across the 15 included studies, HRM was consistently evaluated as a molecular method capable of detecting *Plasmodium* infections and differentiating species using melting temperature profiles and curve-based interpretation. The evidence indicates that HRM has strong diagnostic potential, particularly when compared with microscopy and rapid diagnostic tests, and may provide additional value in settings where submicroscopic or mixed infections are epidemiologically important.

The included studies showed that HRM has been applied across diverse epidemiological contexts, including endemic community settings, asymptomatic surveillance populations, imported malaria cases, and laboratory-based assay validation studies. This diversity reflects the broad potential application of HRM, but also highlights substantial methodological heterogeneity. Differences in study design, sample type, molecular target, comparator method, and diagnostic outcome reporting limited direct comparability across studies and supported a narrative rather than a quantitative synthesis.

### 4.2. Interpretation of Diagnostic Performance

The findings suggest that HRM can achieve high sensitivity and specificity for malaria detection when evaluated against molecular reference methods. Reported sensitivity ranged from 91.76% to 100%, while specificity generally exceeded 92% and reached 100% in several studies. High agreement with nested PCR, LAMP, TaqMan qPCR, and sequencing further supports the analytical validity of HRM as a molecular diagnostic approach [8,11,13,28]. These findings are particularly relevant because HRM combines amplification and post-amplification analysis in a closed-tube format, reducing the risk of contamination and avoiding gel electrophoresis or sequencing for routine interpretation.

Nevertheless, diagnostic performance was not uniform across studies. Variability was influenced by sample source, DNA quality, parasite density, molecular target, and reference standard. For example, HRM performed differently when applied to red blood cell pellets versus archived Giemsa-stained blood smears, suggesting that pre-analytical factors can substantially affect assay sensitivity [10]. False negatives were occasionally reported and were attributed in some cases to DNA degradation or loss during storage and extraction [22]. These findings underscore that HRM performance depends not only on assay design but also on specimen handling, DNA extraction efficiency, and quality assurance procedures.

### 4.3. HRM for Species Differentiation and Mixed Infection Detection

A major strength of HRM identified in this review is its ability to differentiate *Plasmodium* species using species-specific melting profiles. Several studies reported successful differentiation of major human-infecting species, including *P. falciparum*, *P. vivax*, *P. malariae*, *P. ovale*, and *P. knowlesi* [8,12,13]. HRM was also used to discriminate closely related *P. ovale* subspecies, namely *P. ovale curtisi* and *P. ovale wallikeri*, indicating that the method can support fine-scale species or subspecies identification when appropriately designed [9,16].

The ability to detect mixed infections is particularly important in malaria-endemic areas, where microscopy may underestimate species diversity or fail to detect minority parasite populations. Several studies showed that HRM could identify mixed infections involving *P. falciparum*, *P. vivax*, *P. malariae*, and *P. ovale* that were missed or underestimated by microscopy [7,23,24,25]. The hexaplex HRM assay further demonstrated the ability to detect minority species in artificial mixtures at ratios as low as 1:1000, suggesting that optimized multiplex HRM platforms may be useful for identifying complex parasite populations [13].

However, HRM-based species differentiation also has important limitations. Some studies reported narrow melting-temperature differences between species or subspecies, sometimes as small as approximately 0.2 °C, which could compromise interpretation if thermal resolution is insufficient or if the assay is implemented outside specialized laboratory settings [11,16]. In addition, multi-copy 18S rRNA sequence variation within *P. falciparum* may generate double melting peaks that mimic mixed infection profiles, creating a risk of misclassification [9,10]. Therefore, while HRM is promising for species differentiation, its accuracy depends heavily on assay optimization, target selection, platform calibration, and operator expertise.

### 4.4. Value for Low-Parasitemia and Submicroscopic Malaria Detection

The evidence reviewed suggests that HRM may be particularly valuable for detecting low-parasitemia and submicroscopic malaria. Several studies reported limits of detection at or near 1 parasite/µL or a few DNA copies per microliter, depending on the target gene and platform [8,12,13,16]. This level of analytical sensitivity is relevant in malaria elimination settings, where asymptomatic and low-density infections may sustain transmission while remaining undetected by microscopy or RDTs.

Field-based studies further support this potential. HRM detected a higher prevalence of asymptomatic malaria than microscopy in Afghanistan and identified infections in Iran that were missed by both microscopy and RDTs [24,26]. Similarly, HRM detected *Plasmodium* DNA in microscopy-negative specimens from Ugandan children, confirming its utility for identifying submicroscopic carriage [10]. These findings suggest that HRM could support active surveillance, especially in pre-elimination or low-transmission areas where conventional diagnostic tools may underestimate the true parasite reservoir.

However, the evidence remains insufficient to conclude that HRM should be used routinely for population-level screening. The included studies varied in sample size, study population, sampling strategy, and comparator method. Some studies were designed primarily for assay development rather than field implementation, while others evaluated imported malaria or archived specimens. Therefore, the role of HRM in surveillance programs should be further evaluated through prospective studies in endemic settings with standardized sampling, reference testing, and operational outcome measurement.

### 4.5. Analytical and Operational Implications

From an operational perspective, HRM offers several advantages over conventional PCR-based methods. Its closed-tube format reduces amplicon contamination, eliminates gel electrophoresis, and simplifies workflow [7,8,11,13]. Reported turnaround times of approximately 1.25–2 h were shorter than typical nested-PCR workflows, and the use of intercalating dyes may reduce reagent costs compared with probe-based qPCR or sequencing-dependent confirmation [9,12,13].

However, none of the included studies evaluated a commercially standardized HRM-based malaria diagnostic kit; current applications were laboratory-developed assays that varied in targets, primers, reagents, platforms, and interpretation criteria [13,22,28]. HRM also requires a compatible real-time PCR instrument with precise thermal control and high-resolution fluorescence acquisition, as well as dedicated software and trained personnel to distinguish subtle melting-temperature and curve-profile differences [10,11,16]. These requirements limit inter-laboratory reproducibility and routine use in peripheral facilities. HRM is therefore better positioned for reference laboratories and surveillance platforms than as a true point-of-care test.

### 4.6. Methodological Heterogeneity and Implications for Evidence Synthesis

A key finding of this scoping review is the substantial heterogeneity across included studies. HRM assays targeted different genomic regions, including 18S rRNA [7,8,9,10,11,13,24,26], mitochondrial genes [12,13], clpC [16], varATS [28], pfcrt [22], and Pfk13/kelch13 [22,23]. While 18S rRNA was the most frequently used target, other targets were selected to improve species resolution, sensitivity, or resistance marker detection. This diversity illustrates the flexibility of HRM but also complicates comparison across studies.

Heterogeneity was also observed in comparator methods. Some studies used microscopy or RDTs, while others used nested PCR, LAMP, TaqMan qPCR, sequencing, or combinations of these methods. Because microscopy and RDTs are less sensitive for low-density infections than molecular assays, studies that use these methods as comparators may yield different estimates of HRM performance than studies that use qPCR or sequencing. Sample types also varied widely, including whole blood, dried blood spots, FTA cards, archived smears, and red blood cell pellets [8,9,10,13,22,23,24,26,27,28]. These differences directly influence DNA yield and assay sensitivity. As a result, pooled estimation of diagnostic accuracy would not be appropriate without further standardization of assay design, reference standards, and outcome reporting.

### 4.7. Implications for Malaria Diagnosis and Surveillance

The findings of this review suggest that HRM may occupy an intermediate position between conventional molecular diagnostics and field-based screening tools. Compared with microscopy and RDTs, HRM offers improved sensitivity and species-level discrimination. Compared with sequencing or probe-based qPCR, HRM may offer a less expensive and faster alternative for laboratories equipped with compatible real-time PCR platforms. This positions HRM as a potentially useful tool for reference laboratories, epidemiological surveillance, outbreak investigation, imported malaria diagnosis, and confirmation of mixed or non-*falciparum* infections. By improving the detection of infections that conventional diagnostic tools may miss, HRM-based approaches could contribute to malaria control and elimination efforts aligned with Sustainable Development Goal 3.3, which targets the end of malaria and other communicable disease epidemics.

For malaria elimination programs, HRM may be particularly useful in identifying hidden parasite reservoirs, especially asymptomatic, submicroscopic, or mixed infections. This is relevant for low-transmission settings where residual transmission may persist despite negative microscopy or RDT results. However, before HRM can be recommended for routine surveillance or programmatic implementation, future studies need to establish standardized protocols, validate performance in large prospective cohorts, and assess cost-effectiveness in real-world endemic settings.

### 4.8. Evidence Gaps and Future Research Priorities

This review identified several important evidence gaps. First, many HRM assays were evaluated in small sample sets or retrospective collections, limiting confidence in their performance in routine clinical practice. Larger prospective diagnostic accuracy studies are needed across diverse endemic settings, particularly in LMICs where malaria burden and diagnostic constraints are greatest. Second, non-*falciparum* species remain underrepresented in several validation datasets. Further studies should intentionally include *P. vivax*, *P. malariae*, *P. ovale*, and *P. knowlesi*, especially in co-endemic regions.

Third, natural mixed infections require more robust evaluation. Although artificial mixing experiments demonstrate analytical capacity, they may not fully represent the complexity of clinical coinfections, where parasite densities, DNA quality, and species ratios vary widely. Fourth, standardization of HRM targets, thermal profiles, melting temperature thresholds, and reporting formats is urgently needed. Without standardization, comparison across platforms and laboratories will remain difficult. Finally, operational research should evaluate turnaround time, cost per test, training requirements, quality assurance, and feasibility of implementation in endemic settings. No included study directly established HRM as a point-of-care diagnostic approach; therefore, claims regarding point-of-care applicability should remain cautious unless portable or field-adapted HRM platforms are validated.

Beyond these gaps, translation into routine practice will require harmonized targets, thermal profiles, reporting criteria, and multicentre clinical validation. Development of commercially standardized kits, external quality-assurance schemes, compact HRM-capable platforms, and automated melting-curve classification could improve reproducibility and reduce operator dependence. Implementation studies should also assess cost-effectiveness, workflow integration, and feasibility in malaria-endemic LMIC settings before HRM is incorporated into routine surveillance or elimination programmes [5,11,12,13,29].

### 4.9. Strengths and Limitations

This scoping review provides a structured synthesis of HRM applications in malaria diagnosis and maps the evidence across diagnostic performance, species differentiation, mixed-infection detection, low-parasitemia detection, and operational considerations. The review highlights both the promise and limitations of HRM, while identifying methodological heterogeneity and research gaps that should guide future diagnostic studies.

Several limitations should be acknowledged. As a scoping review, the objective was to map the extent and nature of evidence rather than to produce pooled diagnostic accuracy estimates. The included studies varied substantially in design, sample type, molecular target, comparator method, and reporting format, limiting quantitative synthesis. In addition, the quality and completeness of reporting differed across studies; some did not report sensitivity, specificity, limit of detection, or operational metrics. These limitations reflect the current state of the literature and reinforce the need for standardized diagnostic evaluation frameworks for HRM-based malaria assays.

## 5. Conclusions

This scoping review mapped the current evidence on the application of high-resolution melting (HRM) analysis for the molecular diagnosis of malaria across diverse epidemiological settings and laboratory protocols. The included studies consistently reported that HRM can accurately differentiate Plasmodium species and detect mixed-species and low-parasitemia infections, highlighting its potential as a valuable molecular tool for confirmatory diagnosis and malaria surveillance.

However, the available evidence remains methodologically heterogeneous, with substantial variation in molecular targets, assay designs, sample types, reference standards, and outcome reporting. Consequently, the findings should be interpreted as a synthesis of reported evidence rather than a consolidated assessment of diagnostic performance. In addition, the absence of commercially standardized HRM-based malaria diagnostic assays, together with the requirement for specialized real-time PCR instruments, dedicated melting curve analysis software, and experienced personnel, currently limits broader clinical implementation.

Future research should prioritize assay standardization, multicenter clinical validation, and the development of commercially available HRM-based diagnostic platforms to improve reproducibility and facilitate routine implementation. Such advances could strengthen the role of HRM in malaria surveillance and support evidence-based strategies for malaria control and elimination, particularly in endemic low- and middle-income countries.

## Figures and Tables

**Table 2:** Summary of Diagnostic Performance of HRM.

Author (Year)	Comparator	Sensitivity	Specificity	Agreement/Accuracy	Limit of Detection	Key Finding
[16]	Nested PCR	NR	100%	100% concordance	8.90–9.61 copies/µL	Snapback HRM improved *P. ovale* subspecies discrimination
[24]	Microscopy	100%	92.7%	Accuracy 92.9%; kappa 0.302	NR	Detected asymptomatic and mixed infections missed by microscopy
[28]	LAMP	98.1%	100%	Kappa 0.97	NR	In-house HRM showed excellent concordance with LAMP
[8]	Nested PCR, microscopy	NR	NR	Kappa 0.739 vs. nested PCR	~6 copies/µL	Improved resolution of mixed infections
[12]	Nested PCR	NR	98.2%	98.2% agreement	21.47–46.43 copies/µL	Differentiated human *Plasmodium* species using cox1-HRM
[10]	Microscopy	93.0% smear; 100% pellet	96.7% smear; 94.0% pellet	NR	NR	Red cell pellets provided higher sensitivity than archived smears
[13]	qPCR, sequencing	91.76%	98.04%	Kappa 1.00	2.35–3.31 copies/µL	Hexaplex HRM detected minority species at 1:1000 ratio
[9]	Nested PCR, TaqMan qPCR	97.0%	100%	93.6% agreement	1 parasite/µL	Discriminated *P. ovale curtisi* and *P. ovale wallikeri*
[11]	Sequencing, nested PCR	100%	99.31%	Kappa 0.999 vs. sequencing	NR	Detected low-parasitemia *P. falciparum* missed by nested PCR

NR: not reported.

**Table 3:** Thematic Evidence Map of HRM for Malaria Diagnosis.

Main Theme	Studies Supporting the Theme	Key Contribution
Malaria detection	[7,8,9,10,11,12,13,16,22,23,24,25,26,27,28]	HRM was evaluated across all studies as a molecular detection or confirmation method
Species differentiation	[7,8,9,10,11,12,13,16,24,26,28]	HRM differentiated major human *Plasmodium* species using melting profiles
*P. ovale* subspecies differentiation	[9,12,16]	HRM distinguished *P. ovale curtisi* and *P. ovale wallikeri*
Mixed infection detection	[7,8,9,12,13,23,24,25,28]	HRM detected natural or artificial mixed infections, including minority species
Low-parasitemia/submicroscopic detection	[8,9,10,11,12,13,16,23,24,26,27,28]	HRM identified infections missed by microscopy, RDTs, nested PCR, or commercial assays
Treatment monitoring	[27]	HRM detected persistent parasite DNA after treatment and avoided RDT false positivity
Resistance marker profiling	[22,23]	HRM was used for pfcrt and Pfk13/kelch13-related molecular surveillance
Operational advantages	[7,8,9,11,12,13,28]	HRM offered closed-tube workflow, reduced contamination risk, and shorter turnaround time
Implementation constraints	[11,12,13,16]	Specialized equipment and technical expertise limited field and point-of-care applicability

**Table 4:** Evidence Gaps Identified Across Included Studies.

Evidence Gap	Supporting Observation	Implication
Limited large-scale clinical validation	Some assays used small cohorts of 33–41 samples or retrospective archives [8,10,16]	Larger prospective validation studies are needed
Underrepresentation of non-*falciparum* species	*P. malariae*, *P. ovale*, and *P. knowlesi* were often present in small numbers [7,11,13]	More studies are needed in co-endemic regions
Limited validation of natural mixed infections	Some assays relied on artificial mixtures rather than clinical mixed infections [9,11,13]	Algorithms should be validated using natural mixed infections
Lack of standardized HRM targets	Studies used 18S rRNA, mitochondrial genes, clpC, varATS, pfcrt, and Pfk13/kelch13 across different platforms [12,13,16,22,28]	Consensus targets and reporting standards are needed
Melting curve interpretation challenges	Narrow Tm differences and double-peak profiles may complicate interpretation [9,10,11,16]	Standardized interpretation criteria are needed
Unclear point-of-care applicability	HRM requires specialized real-time PCR equipment and trained personnel [11,16,26]	Field-adapted platforms and operational studies are required

## Data Availability

All data supporting the findings of this study are provided within the article and its Supplementary Materials.

## Supplementary Materials

The following supporting information can be downloaded at: https://www.mdpi.com/article/10.3390/pathogens15080815/s1, Table S1: Database-specific search strategies. The table presents the search strategies, applied limits, and numbers of records retrieved from PubMed and Scopus; Table S2: Molecular markers evaluated in HRM-based malaria studies. The table summarizes the genomic location, supporting studies, primary application, and main analytical considerations of each molecular marker.

## Abbreviations

The following abbreviations are used in this manuscript:

ACT	Artemisinin-based Combination Therapy
cox1	Cytochrome c Oxidase Subunit 1
Ct	Cycle Threshold
DBS	Dried Blood Spot
DNA	Deoxyribonucleic Acid
EDTA	Ethylenediaminetetraacetic Acid
HRM	High-Resolution Melting
LAMP	Loop-Mediated Isothermal Amplification
LMICs	Low- and Middle-Income Countries
LOD	Limit of Detection
NGS	Next-Generation Sequencing
NPV	Negative Predictive Value
OSF	Open Science Framework
PCR	Polymerase Chain Reaction
PEO	Population, Exposure, Outcome
Pf	*Plasmodium falciparum*
Pfk13/kelch13	*Plasmodium falciparum* Kelch 13
pfcrt	*Plasmodium falciparum* Chloroquine Resistance Transporter
Pm	*Plasmodium malariae*
POC	Point-of-Care
Po	*Plasmodium ovale*
Poc	*Plasmodium ovale curtisi*
Pow	*Plasmodium ovale wallikeri*
PPV	Positive Predictive Value
PRISMA	Preferred Reporting Items for Systematic Reviews and Meta-Analyses
PRISMA-ScR	Preferred Reporting Items for Systematic Reviews and Meta-Analyses Extension for Scoping Reviews
Pv	*Plasmodium vivax*
qPCR	Quantitative Polymerase Chain Reaction
RDT	Rapid Diagnostic Test
RNA	Ribonucleic Acid
rRNA	Ribosomal Ribonucleic Acid
SDGs	Sustainable Development Goals
SNP	Single-Nucleotide Polymorphism
Tm	Melting Temperature
varATS	Acidic Terminal Sequence of the *var* Gene Family
WHO	World Health Organization
